# Effects of Aromatherapy Combined with Music Therapy on Anxiety, Stress, and Fundamental Nursing Skills in Nursing Students: A Randomized Controlled Trial

**DOI:** 10.3390/ijerph16214185

**Published:** 2019-10-29

**Authors:** Hae Kyoung Son, Wi-Young So, Myoungsuk Kim

**Affiliations:** 1 College of Nursing, Eulji University, Gyeonggi-Do 13135, Korea; sonhk@eulji.ac.kr; 2 Sports and Health Care Major, College of Humanities and Arts, Korea National University of Transportation, Chungju-si 27469, Korea; 3 College of Nursing, Kangwon National University, Gangwon-Do 24341, Korea

**Keywords:** anxiety, aromatherapy, music therapy, nursing skills, nursing students

## Abstract

Purpose: Nursing students often experience anxiety and stress when taking exams that test their fundamental nursing skills. Complementary alternative methods, such as aromatherapy and music therapy, have effectively alleviated such negative emotions among nursing students. However, few studies have examined the effects of combined therapy interventions or compared the effects of different interventions. This study identified the individual and combined effects of aromatherapy and music therapy on test anxiety, state anxiety, stress, and fundamental nursing skills among nursing students in Korea. Methods: A double-blinded, randomized, controlled trial design was used. The study was conducted at the nursing college at Sungshin Women’s University, Seoul, Republic of Korea. Ninety-eight sophomore female nursing students participated in the study. Subjects were randomly categorized under three groups: aromatherapy (*n* = 32), music therapy (*n* = 32), and aromatherapy combined with music therapy (*n* = 34). Aromatherapy was carried out through the inhalation method using an aroma lamp and three drops of *Origanum majorana* and *Citrus sinensis*. Music therapy was carried out using Beethoven’s *Moonlight Sonata*. Twenty-minute interventions were performed in separate rooms before an exam was administered. Data were collected through self-report questionnaires, including demographics, test anxiety, state anxiety, and stress. Participants’ Foley catheterization skill was likewise evaluated. Results: Aromatherapy combined with music therapy had a significant effect on test anxiety (F = 4.29, *p* = 0.016), state anxiety (F = 4.77, *p* = 0.011), stress (F = 4.62, *p* = 0.012), and performance of fundamental nursing skills (F = 8.04, *p* = 0.001) compared with aromatherapy and music therapy as separate interventions. Conclusions: The results suggest that nursing education that includes aromatherapy combined with music therapy may be effective for improving the performance of fundamental nursing skills and reducing anxiety and stress among nursing students.

## 1. Introduction

Nursing is generally based on clinical practice [1] and clinical practice is based on fundamental nursing skills [2], making it important for nurses-in-training to acquire robust fundamental nursing skills. Thus, nursing science curricula have emphasized the importance of fundamental nursing skills and universities have reinforced the importance of these skills by testing nursing students’ skills regularly. Nursing students often experience anxiety over undergoing tests of fundamental nursing skills; their test anxiety is generally higher than that of students in other health-related majors [3]. Additionally, nursing students are required to take various courses in their major besides fundamental nursing skills. Consequently, they experience higher levels of stress than students in other majors [3,4].

Moreover, nursing students’ anxiety and stress increase the likelihood that they will experience negative physiological and behavioral responses regarding the timing of these tests [4,5], which in turn negatively affects their academic performance [6,7]. Experiencing an appropriate level of anxiety or stress can help someone solve a problem or control a situation [6,8]. However, an excessively high level of anxiety and stress can negatively impact someone’s ability to remember important information–especially information stored in the hippocampus [8,9]. That is, the excessive anxiety and stress experienced by nursing students negatively affect their concentration and, by extension, their test performance. Anxiety and stress are also directly related to academic performance, which is negatively affected by decreased interest or motivation difficulty in identifying or describing negative feelings which impacts anxiety and stress [10]. As a result, nursing students often experience maladjustment to campus life and burnout, which results in them taking time off or experiencing withdrawal [11].

The development and validation of interventions that employ complementary alternative methods to alleviate negative emotions among nursing students have been attracting more and more attention. Among these, aromatherapy is of specific interest. Aromatherapy has been reported to have a significant clinical effect on anxiety, depression, and stress. It enhances relaxation and concentration using essential oils extracted from the flowers, stems, roots, and leaves of plants [12,13,14]. In aromatherapy, odorants with relatively small and volatile molecules are easily and swiftly inhaled into the body through the nose and passed through the blood-brain barrier, directly affecting the central nervous system [13,14,15].

Another of these interventions is music therapy, which influences the neuroendocrine system and the autonomic nervous system. Music therapy, such as listening to calm and slow-tempo music, has been reported to reduce anxiety and facilitate relaxation by affecting the limbic system of the brain, which is primarily responsible for controlling emotions [16,17]. Although several studies have assessed these interventions separately, few studies have considered interventions with combined therapies or have compared the effects of different interventions. One exception is a comparative study [18] that compared music therapy and aromatherapy. It concluded that aromatherapy was more effective in soothing anxiety among dental patients and lowering their diastolic blood pressure and respiratory rate. In addition, another study [19] found that combining different types of interventions, such as music therapy, aromatherapy, and meditation, effectively relieved stress and fostered relaxation through a synergistic effect.

Therefore, this study compared the effects of interventions which have been proven effective in addressing anxiety and stress among nursing students who experience extreme anxiety and stress and likewise assessed the effectiveness of a combined intervention. This study is expected to help identify effective interventions to relieve negative emotions related to tests, thereby improving nursing students’ overall academic performance and mental health.

## 2. Materials and Methods

### 2.1. Subjects and Setting

A double-blinded, randomized, controlled trial design was used. Subjects were sophomore students of the nursing college at Sungshin Women’s University, Seoul, Republic of Korea. Subjects were scheduled to undergo a fundamental nursing skill performance test. Individuals who were: allergic to essential oils or had hearing impairments that prevented them from receiving music therapy; participating in other relaxation interventions that affected their anxiety and stress; taking medications for depression or anxiety that could affect stress and anxiety levels; or diagnosed with a psychiatric disorder or known to struggle with substance abuse were excluded from the study.

The sample size was set at a significance level of 0.05, power of 0.80, and an effect size of 0.40, which was extracted from a previous study [13] that researched aromatherapy with students. We calculated the minimum number of subjects required for each of the three groups (aromatherapy, music therapy, and aromatherapy combined with music therapy) for one-way analysis of variance as 27 per group, using G * Power software (G * Power 3.1.7, Heinrich-Heine-University, Düsseldorf, Germany).

### 2.2. Enrollment, Randomization, and Blinding

Data were collected from October 1 to November 10, 2017. Subjects were given verbal and written explanations of the study approximately one week before the test. The researchers conducted the preliminary survey after obtaining written consent from the subjects, with a total of 107 subjects taking part in the survey.

Randomization was conducted by a research assistant who was not associated with the study using the Research Randomizer (https://www.randomizer.org/). A card with a randomly assigned number indicating an assigned group was put into an opaque envelope. The research assistant opened the envelope for each subject in the order of their arrival and confirmed their assigned group. The double-blinded method was used so that neither the evaluators nor the subjects knew the assigned group.

**Figure 1:**
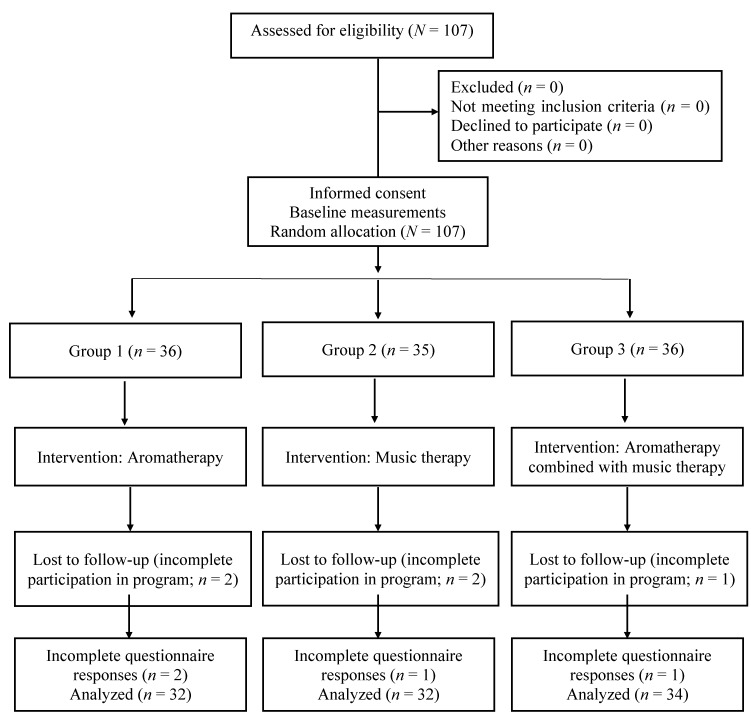
Research flow diagram.

The subjects who wanted to participate in the study because they had been notified that the department was recruiting research assistants were screened for suitability based on the inclusion criteria. All 107 eligible participants were enrolled (Figure 1). After completing the enrollment and baseline measurements, the participants were randomly assigned to the aromatherapy group (*n* = 36), music therapy group (*n* = 35), or aromatherapy combined with music therapy group (*n* = 36). Two subjects did not fully participate in the intervention in the aromatherapy group, two in the music therapy group, and one in the aromatherapy combined with music therapy group. Additionally, all items in the questionnaire were left unchecked by two subjects in the aromatherapy group, one in the music therapy group, and one in the aromatherapy combined with music therapy group. Excluding those nine subjects, 32 students participated in aromatherapy, 32 participated in music therapy, and 34 participated in aromatherapy combined with music therapy. Therefore, this study analyzed the data of 98 subjects in total.

### 2.3. Interventions

The essential oils utilized in the intervention were selected after consultation with a licensed aromatherapy expert who has been teaching an aromatherapy training course for more than 16 years, together with a review of previous literature. Based on this consultation and review, we selected *Origanum majorana* (sweet marjoram), which is effective in relieving stress, soothing, and emotional relaxation, and *Citrus sinensis* (orange), which is effective in reducing nervousness and stress [20]. Based on results of previous studies which found that combining two to three types of essential oils is more effective for relieving stress and relaxation than a single type of essential oil [15], three drops of *Origanum majorana* and *Citrus sinensis* were mixed together at a ratio of 1:1 and diffused into the air using a lamp so that subjects could inhale the odorants. The diffused essential oil was inhaled and spread through the body and reached peak levels within 20 min [14,15]. Thus, aromatherapy was conducted for 20 min in this study.

For music therapy, Beethoven’s *Moonlight Sonata* [16] was used, as it has been proven effective in relieving test anxiety and state anxiety by improving listeners’ stress levels, blood pressure, pulse rate, and body temperature. Music was played continuously in a space equipped with a sound system so that subjects could listen to it casually, without earphones. Each intervention session was conducted for 20 min [16]. The results of a previous study found that listening to soft music for five min before taking a test reduced anxiety levels, improved blood pressure and pulse rate, and improved the academic performance of college students [21]. Based on previous results, the subjects in this study were naturally exposed to music for the same 20-minute intervention time as the subjects in the aromatherapy group.

The interventions developed for each group in this study are presented in Table 1. Each subject was told to arrive at the intervention site 40 min prior to the scheduled test. Once each subject arrived, they were randomly assigned to a group by a research assistant. The subject was then immediately guided by the research assistant to the intervention room to prevent them from discussing the intervention with other subjects.

Each subject received the assigned intervention for 20 min in a separate room before the test of fundamental nursing skills. Each intervention space had the same temperature of 26–27 °C and medium-level lighting to eliminate factors other than the intervention. Additionally, roller shades were used to control the lighting and prevent subjects from looking out of the window. Three research assistants were given prior training via the manual and allocated to each intervention space. The research assistants were responsible for preparing the questionnaire, checking the intervention time and environment, and giving subjects notice about starting the test. For aromatherapy, different types of aroma oils were mixed by a researcher with an aromatherapy license according to the opinions of an aromatherapy expert. The research assistants who were assigned to groups receiving aromatherapy checked whether the aroma oil had been diffused into the air. They replenished the water in the lamp when it was depleted and refreshed the prepared mixture of aroma oil. The research assistants in the groups receiving music therapy ensured that music played continuously and insured that sound fidelity was adequate.

After the intervention, each subject’s proficiency in fundamental nursing skills was evaluated by three professors (namely Foley catheterization) in an environment separate from the intervention area. The test required 10 min for each subject. The test was conducted under consistent physical conditions, including temperature, lighting, and prepared items (model for Foley catheterization, catheter, and so forth). To reduce inconsistencies between evaluators, the evaluators used a structured score-card provided by the Korea Accreditation Board of Nursing Education, which clearly presents detailed assessment items and guidelines for evaluators. To reduce evaluator-specific bias, the assessors rated subjects without knowing their intervention group. Subjects were immediately guided by research assistants from the intervention area to the testing area, and subjects returned home immediately after the test. Therefore, contact between subjects during the experiment was prevented.

### 2.4. Assessments

#### 2.4.1. General Characteristics

Subjects’ characteristics, including religion, subjective health status, satisfaction with nursing, overall grade point average in the prior semester, music preferene, and demand for aromatherapy were considered.

#### 2.4.2. Test Anxiety

Test anxiety was measured using the Korean version of the Revised Test Anxiety Scale developed by Benson and El-Zahhar [22] and translated and validated by Cho [23]. The scale consists of four sub-factors and a total of 20 questions, with five questions on nervousness, six on concern, five on physical symptoms, and four on error-free tests. Each question is answered on a four-point Likert scale, with a score of one denoting “I rarely feel it,” and a score of four denoting “I almost always feel it.” The higher the score, the higher the level of test anxiety. In Cho’s study [23], Cronbach’s α was 0.90; in the current study, it was 0.93.

#### 2.4.3. State Anxiety

State anxiety was measured using the Korean version of the Spielberger State Anxiety Inventory-Y developed by Hahn, Lee, and Chon [24]. Questions are answered on a four-point Likert scale, with a score of one denoting “It is completely untrue,” and a score of four denoting “It is absolutely true.” Scores are totaled to give a possible range of 20 to 80 points. Higher scores denote higher levels of anxiety. The scale was purchased from the website of Hakjisa Publisher, Inc., which owns the copyright for the Korean version of the instrument. At the time of translation, the instrument’s reliability, as represented by Cronbach’s α, was 0.92 [24]. It was 0.89 in this study.

#### 2.4.4. Stress

Stress was measured using a numeric rating score (NRS) developed by Cohen, Kamarck, and Mermelstein [25], which provides a subjective score for stress. This instrument allowed participating nursing students to indicate their perceived level of stress ranging from 0 (“I have no stress at all”) to 10 (“I have a very high level of stress”). At the time of translation, the reliability of the instrument, as represented by Cronbach’s α, was 0.76 [26]. It was 0.84 in this study.

#### 2.4.5. Performance of Nursing Skills

To measure nursing skills, we converted items from the indwelling catheter insertion skill test into a checklist format. The specific skill test was the Essential Fundamental Nursing Skill Assessment Items Protocol (version 4.1), developed by the Korea Accreditation Board of Nursing [27]. Each item was checked as “performed” or “not performed,” and a composite score was generated based on a 100-point scale; the higher the composite score, the higher the level of the subjects’ nursing skill performance.

### 2.5. Ethical Considerations

This study was approved by the institutional review board (No. SSWUIRB 2017-055) of Sungshin Women’s University and was conducted with participants who met the inclusion criteria. Participants were given a prior explanation of the purpose and methods of the study using a structured research guide. Written informed consent was obtained from each participant. The researchers informed the participants that the collected data would not be used for any other purpose and that anonymity, confidentiality, and destruction of the data after the study were guaranteed. Furthermore, the researchers respected the voluntary participation and intentions of participants and informed the participants that they could withdraw from the study at any time.

### 2.6. Statistical Analysis

IBM SPSS Statistics ver. 21.0 software (IBM Corp., Armonk, NY, USA) was used for data analysis. Homogeneity tests for the general characteristics of each group consisted of the χ^2^-test and Fisher’s exact test, and the normality of the dependent variables was confirmed using the Shapiro–Wilk test. One-way analysis of variance was conducted on the dependent variables to determine the effects of the experimental intervention. Statistical significance was defined as *p* < 0.05.

## 3. Results

Subjects’ general characteristics are presented in Table 2. There were no significant differences in these characteristics among the three groups (*p* > 0.05). Additionally, baseline scores of the main variables (test anxiety, state anxiety, and stress) did not differ among groups before the intervention.

The effects of the interventions are presented in Table 3. There was a significant difference among all three groups for test anxiety (F = 4.29, *p* = 0.016), state anxiety (F = 4.77, *p* = 0.011), stress (F = 4.62, *p* = 0.012), and fundamental nursing skill performance (F = 8.04, *p* = 0.001). Aromatherapy combined with music therapy was associated with a significant decrease in test anxiety, state anxiety, stress, and increased subjects’ fundamental nursing skill performance, as compared with the separate intervention groups. There was no significant difference between the separate intervention groups.

## 4. Discussion

This study confirmed the effects of aromatherapy and music therapy when applied separately and when combined. Additionally, this randomized experimental study used interventions with high reproducibility, which could be utilized by nursing students before a test. Aromatherapy combined with music therapy was associated with a significant decrease in test anxiety, state anxiety, and stress, and significantly increased students’ fundamental nursing skill performance compared with the other separate intervention groups. 

Excessive test anxiety and stress negatively affect academic performance [6,7] and have a negative impact on students’ ability to perform academic tasks [8,9]. Aromatherapy has been proven to be an effective strategy to lower students’ level of distraction caused by test anxiety and stress [3]. Additionally, music therapy has been confirmed to be an effective diversion therapy to relieve anxiety and foster relaxation under circumstances that cause extreme nervousness and stress [16,21,28]. A study of the effects of interventions that combined aromatherapy, music therapy, meditation, and other methods of relieving anxiety and stress confirmed that combination therapy was more effective than individual therapies in relieving anxiety and stress [19]. The combined use of two types of interventions in this study was associated with significantly improved performance of fundamental nursing skills and reduced stress and test anxiety through a synergistic effect. This indicates that aromatherapy, combined with music therapy, is an effective intervention for improving performance among nursing students.

Additionally, although there was a significant difference between aromatherapy combined with music therapy and single intervention modalities, there was no significant difference between separate interventions of aromatherapy and music therapy. This is consistent with previous studies [3,21,28], wherein no significant difference was observed between the separate interventions because both aromatherapy and music therapy reduce negative emotions, such as anxiety or stress, among subjects who perform demanding cognitive tasks.

The aromatherapy techniques in this study were based on previous studies [14,15]. The essential oil was diffused through the lamp in the intervention room, inhaled, and spread throughout the body. This method minimized the bias that could have been caused by applying individual interventions to the subjects in different rooms. Moreover, essential oils reach their maximum level in the body within 20 min of inhalation and directly affect the brain [14,15]. Therefore, it was considered appropriate to provide aromatherapy for 20 min. This intervention method and duration can be applied to nursing students preparing for tests because the method does not impose a heavy burden on the intervention recipient.

Music therapy was provided by selecting music that has been found to be effective for relieving test anxiety in previous studies [16,19,21]. The music was played in the intervention area prior to the test so that subjects could listen to music under normal circumstances. Beethoven’s *Moonlight Sonata* was chosen based on previous studies [16,29] that found music effective for relieving anxiety, and considering the results of a preliminary survey which revealed that the subjects preferred classical music. Additionally, in a previous study, subjects showed increased positive emotions, decreased anxiety [28,30], and improved performance when they performed tasks [28] while listening to their preferred music. Therefore, it is important to consider music preferences when providing music therapy.

For the combined therapy environment, this study tried to mitigate the influence of external variables by regulating the intervention room’s temperature and lighting. It is assumed that effective use of these therapies requires a suitable physical space to comfortably undergo aroma and music therapy. This suggests that the findings of this study can be applied to real-life situations, provided that students can reserve the necessary space to pursue these therapies. If it is not possible to prepare such an intervention room, it may be better to control the environment through other means because various external variables will be affected by the surrounding environment.

This study is of particular utility because it explored various aspects of the effects of aromatherapy combined with music therapy and also compared the effects of separate interventions. Further, this study used a double-blinded, randomized method. The results of this study are highly applicable to nursing students preparing for tests, such as tests of fundamental nursing skills, and provides basis for more effective educational interventions for nursing students.

Based on the results of this study, we note the following limitations and provide suggestions accordingly. First, as this study was conducted with sophomores at a nursing college for convenience, it did not consider participants who varied in gender, age, and race. Therefore, researchers should not generalize the results of this study too widely; personal and social factors that can affect test anxiety, stress, and task performance should be considered in future research. Second, while this study used self-report questionnaires to measure anxiety and stress, future research might also measure physiological variables to enhance the objectivity and validity of study results. Finally, the results of this study should be interpreted while considering the possibility of placebo effects caused by positive perceptions of the interventions as reported in the preliminary survey, participants’ intervention preferences, and the guidance given to the participants prior to the intervention.

## 5. Conclusions

This study showed that aromatherapy combined with music therapy had a significant effect on test anxiety, state anxiety, stress, and performance of fundamental nursing skills compared with separate interventions. Based on the results, aromatherapy combined with music therapy is recommended as an intervention for nursing students preparing for tests so that they can achieve the learning objectives of the nursing curriculum. Additionally, this study provided distinctive insights as it examined the effect by combining aroma and music therapy in nursing students who were facing tests of their fundamental nursing skills.

## Figures and Tables

**Table 1:** Intervention process.

Process	Group			Duration(min)
	AG	MG	AMTG	
Baseline measurements	Music preferences, preference for aromatherapy, exclusion criteria			5
Introduction	Program introduction (procedure, pretest, posttest, fundamental nursing skill test)			5
Pretest	Test anxiety, state anxiety, stress			10
Intervention	Method: lamp inhalation Aroma oil: marjoram and orange	Music type: Beethoven’s *Moonlight Sonata*	Method: lamp inhalation Aromaoil: marjoram and orange Music type: Beethoven’s *Moonlight Sonata*	20
Posttest	Test anxiety, state anxiety, stress			5
Fundamental nursing skill test	Fundamental nursing skill performance test			10

AG: aromatherapy group; MTG: music therapy group; AMTG: aromatherapy combined with music therapy group.

**Table 2:** Homogeneity of subjects’ general characteristics and variables among groups.

Characteristics		AG (*n* = 32)	MTG (*n* = 32)	AMTG (*n* = 34)	χ^2^ or F	*p*
Religion	Christianity	8 (25.0)	7 (21.9)	3 (8.8)	6.53	0.340
	Buddhism	2 (6.3)	2 (6.3)	0 (0.0)		
	Catholicism	2(6.3)	3 (9.4)	4 (11.8)		
	None	20 (62.5)	20 (62.5)	27 (79.4)		
Subjective health status	Good	23 (71.9)	25 (78.1)	29 (85.3)	3.18	0.507
	Moderate	8 (25.0)	7 (21.9)	5 (14.7)		
	Poor	1 (3.1)	0 (0.0)	0 (0.0)		
Satisfaction with nursing	Satisfied	19 (59.4)	14 (43.8)	17 (50.0)	4.18	0.354
	Moderate	11 (34.4)	16 (50.0)	17 (50.0)		
	Not satisfied	2 (6.3)	2 (6.3)	0 (0.0)		
Overall GPA in the prior semester	≥ 4.0	1 (3.1)	5 (15.6)	6 (11.9)	9.62	0.128
	3.5–3.99	16 (50.0)	16 (50.0)	20 (58.8)		
	3.0–3.49	10 (31.3)	8 (25.0)	8 (23.5)		
	< 3.0	5 (15.6)	3 (9.4)	0 (0.0)		
Music preference before evaluation	Classical	18 (56.3)	21 (65.6)	19 (55.9)	4.27	0.658
	Ballad	5 (15.6)	6 (18.8)	4 (11.8)		
	Jazz	7 (21.9)	4 (12.5)	6 (17.6)		
	Others	2 (6.3)	1 (3.1)	5 (14.7)		
Demand for aromatherapy	Very needed	16 (50.0)	15 (46.9)	14 (41.2)	6.71	0.293
	Needed	12 (37.5)	16 (50.0)	12 (35.3)		
	Moderately needed	3 (9.4)	1 (3.1)	7 (20.6)		
	Unnecessary	1 (2.9)	0 (0.0)	1 (2.9)		
Test anxiety		42.71 ± 7.85	43.09 ± 12.47	42.32 ± 7.00	0.05	0.946
State anxiety		54.31 ± 10.23	54.43 ± 10.87	55.05 ± 7.18	0.06	0.943
Stress		6.34 ± 2.05	6.81 ± 1.22	6.41 ± 1.04	0.91	0.404

AG: aromatherapy group; MTG: music therapy group; AMTG: aromatherapy combined with music therapy group; Values are mean ± standard deviation or *n* (%); Tested by Fisher’s exact test or analysis of variance.

**Table 3:** Comparisons of the change in scores among groups (*n* = 98).

Variables	Group	Pre-test	Post-test	Difference (pre-post)	F	*p*	Scheffé post-hoc
Test anxiety	AG (*n* = 32)	42.71 ± 7.85	42.21 ± 9.01	0.50 ± 6.68	4.29	0.016*	AMTG > AG, MTG
	MTG (*n* = 32)	43.09 ± 12.47	42.68 ± 10.09	0.41 ± 5.47			
	AMTG (*n* = 34)	42.32 ± 7.00	38.06 ± 6.55	4.26 ± 6.16			
State anxiety	AG (*n* = 32)	54.31 ± 10.23	53.09 ± 8.20	1.22 ± 8.82	4.77	0.011*	AMTG > AG, MTG
	MTG (*n* = 32)	54.43 ± 10.87	52.78 ± 9.34	1.65 ± 7.53			
	AMTG (*n* = 34)	55.05 ± 7.18	48.47 ± 7.69	6.58 ± 7.20			
Stress	AG (*n* = 32)	6.34 ± 2.05	6.31 ± 0.96	0.03 ± 1.84	4.62	0.012*	AMTG > AG, MTG
	MTG (*n* = 32)	6.81 ± 1.22	6.68 ± 1.46	0.12 ± 1.73			
	AMTG (*n* = 34)	6.41 ± 1.04	5.17 ± 1.88	1.24 ± 1.81			
Fundamental nursing skills performance	AG (*n* = 32)		73.37 ± 17.41		8.04	0.001**	AMTG > AG, MTG
	MTG (*n* = 32)		68.90 ± 20.34				
	AMTG (*n* = 34)		84.29 ± 6.96				

AG: aromatherapy group; MTG: music therapy group; AMTG: aromatherapy combined with music therapy group; Values are mean ± standard deviation; * *p* < 0.05 by analysis of variance in difference (pre-post); ** *p* < 0.01 by analysis of variance in post-test.
